# Anomalies of the Teeth

**Published:** 1844-10

**Authors:** 


Dr. Dillingham, in the Boston Medical and Surgical Journal for July 22, reports the following cases of

"*Anomalies of the Teeth.*—The permanent teeth complete their growth at about the age of 21 years. Anomolies, however, sometimes occur—first, in the number of the teeth, and sometimes in an entire deficiency of teeth in the jaw. Mr. Fox mentions a number of cases in which the teeth were entirely deficient. Pliny has mentioned a curious case, which was that of Pyrrhus, King of Epirus, in whom all the crowns were united.

The period at which teeth appear varies much. Instances are recorded of children that have been born with teeth. On the other hand, we have heard of instances where the teeth have not appeared for a number of years. A young child, nine and half months of age, in this town, has twelve teeth—their growth entirely completed. In one instance I inserted four teeth for a lady 40 years of age (two central and two lateral incisors). Three months after they were inserted, she had a complete set of natural teeth.

Another case is at present under my observation. A young lady, 22 years old, called on me some months since, and had a cuspid or eye-tooth put in on plate. A few days ago she called at my office, and requested me to fasten the plate in, as it continually got loose. On inspection, I found a new cuspid making its appearance where it should have been twelve years ago.

